# Determine the Relative Aromaticity of Bilayer Graphyne, Bilayer Graphdiyne, and Bilayer Graphtriyne

**DOI:** 10.3390/molecules30020365

**Published:** 2025-01-17

**Authors:** Gang Zhang, Xinwen Gai, Lulu Sun, Ji Ma

**Affiliations:** College of Science, Liaoning Petrochemical University, Fushun 113001, China; zhanggang0308@163.com (G.Z.); gaixinwen1025@163.com (X.G.)

**Keywords:** aromaticity, molecular orbital analysis, TD-DFT photophysical properties

## Abstract

The electronic structure characteristics of bilayer graphyne, bilayer graphdiyne, and bilayer graphtriyne were systematically studied using molecular orbital (MO) analysis, density of states (DOS), and interaction region indicator (IRI) methods. The delocalization characteristics of the out-of-plane and in-plane π electrons (i.e., π^out^ and π^in^ electrons) of these materials were analyzed using the localized orbital locator (LOL). In addition, their responses to external magnetic fields were investigated through anisotropic induced current density (AICD) and isoscalar chemical shielding surfaces (ICSSs) to compare the induced ring currents and magnetic shielding effects, further exploring the aromaticity of the three bilayer materials. The research results indicate that as the number of alkyne groups increases, the aromaticity of the bilayer graphyne structure gradually weakens. Finally, their photophysical properties were studied through TD-DFT calculations. The results show that they exhibit strong localized excitation characteristics.

## 1. Introduction

Graphyne, as a novel carbon-based material, has attracted widespread attention in materials science in recent years [1]. Graphyne materials consist of two-dimensional structures formed by sp- and sp^2^-hybridized carbon atoms, featuring a unique π-conjugated electron system and high electron mobility. These characteristics give them great potential for applications in electronics, optoelectronics, catalysis, and sensing [2,3]. The study of graphyne originated from the extension of graphene, where graphyne is formed by introducing alkyne groups (-C≡C-) to replace some of the carbon–carbon single bonds, resulting in a lattice structure with vacancies. This not only imparts unique electronic properties to graphyne but also demonstrates excellent performance in mechanical strength, chemical stability, and thermal conductivity [4].

The synthesis of graphyne was first achieved in 2010 by Professor Yu-Liang Li’s research team, who successfully synthesized monolayer graphyne on a copper surface using chemical vapor deposition (CVD) and conducted preliminary characterization of its structure and electronic properties. This study provided the first confirmation of the structural stability and potential application value of graphyne, a novel two-dimensional carbon material, thereby pioneering the field of graphyne material research [5,6]. Subsequently, Zhou et al. further optimized the synthesis of graphyne films using a surface templating method, achieving uniform layer number and structural regularity at the nanoscale [7]. In the study of bilayer graphyne, the number of layers has a significant impact on its aromaticity and electronic properties. Jia et al. found through theoretical calculations that increasing the number of graphyne layers significantly enhances its bandgap characteristics, increasing its potential for applications in optoelectronic devices, such as field-effect transistors. In particular, in the bilayer structure, the enhancement of interlayer π-π interactions leads to a notable improvement in electronic conductivity [8,9]. Lee et al. found through theoretical calculations that bilayer graphyne exhibits excellent carrier mobility and a high on/off ratio, making its performance in field-effect transistors superior to that of monolayer structures [10]. Zhao et al. emphasized the application potential of graphyne in optoelectronic conversion and electrochemistry, particularly in solar cells and photocatalysis, demonstrating the advantages of multilayer graphyne as an efficient optoelectronic material [11].

Aromaticity is a fundamental concept in chemical research, having a profound impact on the structure, stability, and reactivity of numerous molecular systems [12,13,14]. Since the 19th century, this concept has gradually evolved to describe the unique stability of conjugated cyclic π electron systems [15]. Initially, Kekulé proposed the concept of aromaticity based on the resonance structure of the benzene ring to explain its unique chemical stability. Later, Hückel introduced the (4n + 2)π electron rule through molecular orbital theory, providing a quantitative criterion for aromaticity and laying the theoretical foundation for the study of aromaticity in chemistry [16]. Aromatic systems typically resist addition and oxidation reactions but exhibit high reactivity in electrophilic substitution reactions, making them widely applicable in synthesis and catalysis [17].

The development of aromaticity theory has greatly advanced the research of novel carbon-based materials. Due to the high symmetry and conjugated π electron structure of graphyne, it satisfies Hückel’s aromaticity rule and exhibits aromatic properties similar to the benzene ring. As a typical conjugated carbon material, graphyne also demonstrates excellent electronic structure and aromatic characteristics through molecular orbital analysis [18,19,20]. Graphyne is composed of sp- and sp^2^-hybridized carbon atoms, featuring a highly π-conjugated two-dimensional structure and uniform pore distribution. It is considered an efficient conductive material, demonstrating significant application potential in optoelectronics, catalysis, and energy storage [21]. The aromaticity in graphyne is not only dependent on its molecular structure but also closely related to the highly delocalized electrons [22]. Yin et al. successfully prepared hydrogenated graphyne powder through a dehalogenation self-coupling reaction, demonstrating its stability and aromatic characteristics in electrocatalytic applications [23]. In addition, studies have shown that as the number of alkyne chains increases, the aromaticity of bilayer graphyne gradually weakens, and the electronic delocalization characteristics undergo significant changes [24]. Krygowski et al. pointed out that the aromaticity features of the graphyne structure are particularly pronounced [25].

In this study, the electronic structure and aromaticity characteristics of bilayer graphyne and its related structures were systematically investigated through graphical representation and theoretical calculations, revealing their fundamental properties. Research has shown that the aromaticity of bilayer graphyne plays a key role in regulating its optical nonlinearities. Previous studies have indicated that organic conjugated structures with strong aromaticity typically exhibit significant nonlinear optical properties, especially in molecules with special topological structures [26,27]. This study not only reveals the aromaticity characteristics of bilayer graphyne from an electronic structure perspective but also provides an in-depth analysis of its photophysical and nonlinear properties.

## 2. Results

### 2.1. Optimized Structure and Calculation Level

**Figure 1:**
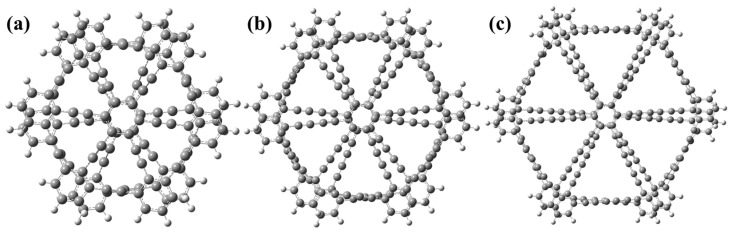
(**a**) The geometric structure of bilayer graphyne was optimized at the ωb97xd/6-311G(d,p) level. (**b**) The geometric structure of bilayer graphdiyne was optimized at the ωb97xd/6-311G(d,p) level. (**c**) The geometric structure of bilayer graphtriyne was optimized at the ωb97xd/6-311G(d,p) level.

At the ωb97xd/6-311G(d,p) level of theory, three types of graphyne structures were optimized. Figure 1a illustrates the structure of bilayer graphyne, which typically exhibits strong interlayer π-π interactions after optimization, with an interlayer distance of approximately 3.4 Å and a twist angle of 13.25°. Figure 1b depicts the structure of bilayer graphdiyne, characterized by a denser pore structure. Its interlayer distance is slightly smaller than that of bilayer graphyne, approximately 3.2 Å, with a twist angle of 12.42°. Figure 1c presents the structure of bilayer graphtriyne, which has the largest interlayer distance of approximately 3.6 Å and a twist angle of 7.23°.

### 2.2. HOMO and LUMO Analysis

**Figure 2:**
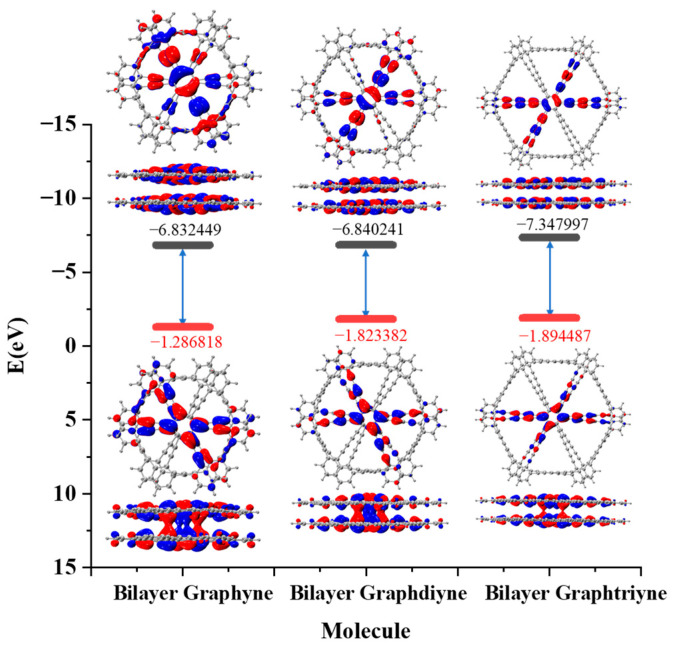
HOMO and LUMO of bilayer graphyne, bilayer graphdiyne, and bilayer graphtriyne, along with the HOMO-LUMO gap. In the HOMO-LUMO isosurface, red represents the negative phase, and blue represents the positive phase.

As shown in Figure 2, the calculated energies of the highest occupied molecular orbital (HOMO) and the lowest unoccupied molecular orbital (LUMO) for bilayer graphyne are −6.83 eV and −1.29 eV, respectively, corresponding to a HOMO-LUMO gap of 5.55 eV. For bilayer graphdiyne, the HOMO and LUMO energies are −6.84 eV and −1.82 eV, with a band gap of 5.02 eV. In the case of bilayer graphtriyne, the HOMO and LUMO energies are −3.45 eV and −1.89 eV, respectively, with the HOMO-LUMO gap reaching 5.45 eV. These results indicate that as the number of acetylene groups increases, the HOMO-LUMO gap first decreases and then increases, suggesting a corresponding change in the ease of electronic transitions. Bilayer graphyne demonstrates the least propensity for transitions, whereas bilayer graphdiyne exhibits the most facile transitions.

### 2.3. Electronic Structure: Molecular Orbital (MO) and Density of States (DOS) Analysis

**Figure 3:**
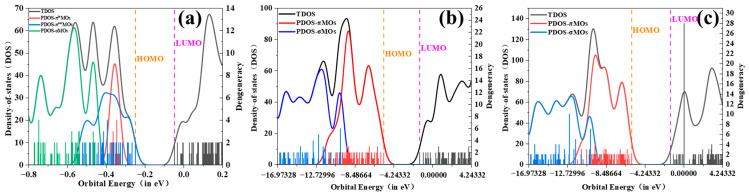
(**a**) PDOS and TDOS curves of bilayer graphyne; (**b**) PDOS and TDOS curves of bilayer graphdiyne; (**c**) PDOS and TDOS curves of bilayer graphtriyne.

The molecular orbital (MO) analysis results show that bilayer graphyne contains three types of valence electron-occupied orbitals: π^in^-MOs, π^out^-MOs, and σ-MOs. In contrast, bilayer graphdiyne and bilayer graphtriyne each have two types of valence electron-occupied orbitals. Figure 3 shows the contribution of all occupied molecular orbitals to the total density of states (DOS) and the contribution of partially occupied molecular orbitals to the projected density of states (PDOS) for each system of bilayer graphyne, bilayer graphdiyne, and bilayer graphtriyne. As shown in Figure 3a, the lower energy range of bilayer graphyne is mainly contributed by σ-MOs. In the energy range from −10 eV to −4.4 eV, the contributions come from σ-MOs, π^in^-MOs, and π^out^-MOs. The region around −0.5 eV to 4.2 eV corresponds to unoccupied orbitals. Figure 3b shows that the lower energy range of bilayer graphdiyne is also dominated by σ-MOs. In the energy range from −17 eV to −3.1 eV, the contributions come from both σ−MOs and π-MOs, while the orbitals around −0.5 eV to 4.2 eV are unoccupied. Figure 3c indicates that the lower energy range of bilayer graphtriyne is primarily contributed by σ-MOs. In the energy range from −17 eV to −3.2 eV, both σ-MOs and π-MOs contribute, and the orbitals around −0.5 eV to 4.2 eV are unoccupied.

### 2.4. Interaction Region Indicator (IRI-π) Analysis

**Figure 4:**
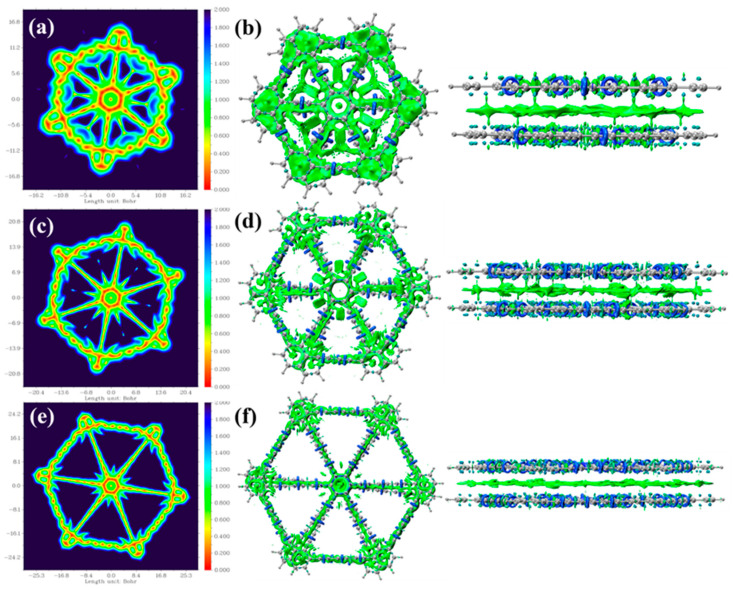
(**a**) Color-filled map 0.5 Å above bilayer graphyne; (**b**) IRI-π isosurface map of bilayer graphyne; (**c**) color-filled map 0.5 Å above bilayer graphdiyne; (**d**) IRI-π isosurface map of bilayer graphdiyne; (**e**) color-filled map 0.5 Å above bilayer graphtriyne; (**f**) IRI-π isosurface map of bilayer graphtriyne. Isosurface: 1 a.u., with the color scale in a.u. The bluer regions on the IRI-π isosurface indicate higher π-electron density, suggesting stronger π-interactions, whereas the greener regions indicate lower π-electron density, implying weaker π-interactions.

The interaction region indicator (IRI) analysis clearly reveals the presence and types of chemical bonds and weak interactions between atomic or molecular fragments [28]. In calculating the IRI-π, only the electron density and its gradient associated with π orbitals are considered, which visually displays the location, type, and strength of interactions within the chemical system. The green isosurface between the two layers of graphyne typically represents the region of non-covalent interactions dominated by van der Waals forces. As shown in Figure 4b,d,f, the C≡C triple bonds in bilayer graphyne, bilayer graphdiyne, and bilayer graphtriyne exhibit blue ring-shaped features on the IRI-π isosurface. Additionally, the C-C bonds in the central benzene ring of bilayer graphtriyne also display a blue coloration, indicating the presence of dual π interactions (π^out^ and π^in^) in this region. The dark blue contours in the C≡C triple bond regions display the highest π electron density, indicating the strongest π effect. The IRI-π isosurfaces of other C-C bonds appear as green rings that extend on both sides of the molecular plane without closing, indicating that these C-C bonds only exhibit a single π interaction (π^out^). Additionally, Figure 4a,c,e present the IRI-π colored maps of bilayer graphyne, bilayer graphdiyne, and bilayer graphtriyne at a plane of 2 Bohr, further illustrating the distribution characteristics of π electrons and providing an intuitive understanding of the electronic distribution and interactions in these materials.

### 2.5. Local Orbital Locator (LOL) Analysis

**Figure 5:**
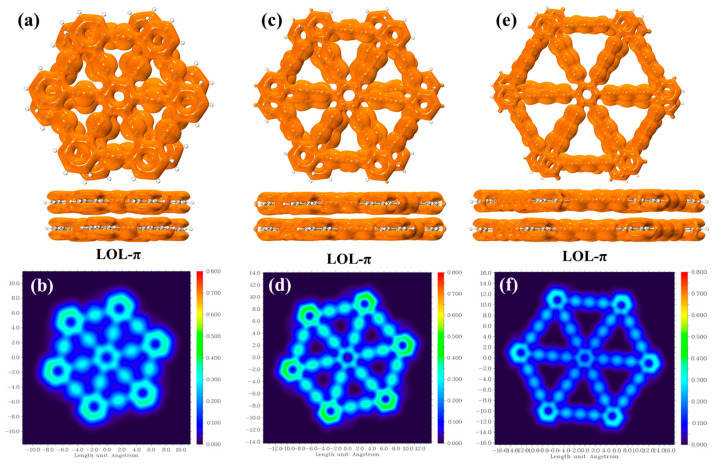
(**a**) LOL-π isosurface at 0.4 Å above the molecule for bilayer graphyne; (**b**) LOL-π color-filled plot at 0.5 Å above the molecule for bilayer graphyne; (**c**) LOL-π isosurface at 0.4 Å above the molecule for bilayer graphdiyne; (**d**) LOL-π color-filled plot at 0.5 Å above the molecule for bilayer graphdiyne; (**e**) LOL-π isosurface at 0.4 Å above the molecule for bilayer graphtriyne; (**f**) LOL-π color-filled plot at 0.5 Å above the molecule for bilayer graphtriyne. Isosurface value: 0.2 a.u., and the color scale is represented in a.u. The transition from blue to green to red indicates an increasing degree of electron delocalization.

In 2020, Schmider et al. proposed a function called the Local Orbital Locator (LOL) to reveal the distribution of localized electrons. A variant of the LOL, called the LOL-π, considers only the contribution of π electrons and exhibits unique advantages in analyzing delocalized bonds and molecular aromaticity [29]. In this study, we calculated the LOL-π for the π molecular orbitals (π-MOs), and the resulting isosurface and color-filled maps are shown in Figure 5. Figure 5a shows that the π-MOs of bilayer graphyne exhibit a well-delocalized overall distribution, with strong electron delocalization in the C≡C triple bonds. In the filled contour map of the LOL-π shown in Figure 5b, significant changes in the color distribution on the C≡C bonds can be observed, reflecting the non-uniformity of electron delocalization. Figure 5c illustrates that the π-MOs of bilayer graphdiyne also display a well-delocalized overall distribution. The corresponding filled contour map of the LOL-π in Figure 5d similarly shows significant color variations on the C≡C bonds, indicating non-uniform electron delocalization. Figure 5e demonstrates a well-delocalized electron channel in the π-MOs of bilayer graphtriyne. In the filled contour map of the LOL-π shown in Figure 5f, significant color changes on the C≡C bonds are still evident, revealing the non-uniformity of electron delocalization. By comparing the isosurface and color-filled map, it can be observed that the connectivity of the isosurface and color-filled map for bilayer graphyne is superior to that of bilayer graphdiyne and bilayer graphtriyne, while the connectivity of bilayer graphtriyne is lower than that of bilayer graphdiyne. This trend indicates that as the number of acetylenic linkages increases, the molecular aromaticity gradually weakens.

### 2.6. Response to External Magnetic Field: Anisotropic Induced Current Density (AICD) Analysis

**Figure 6:**
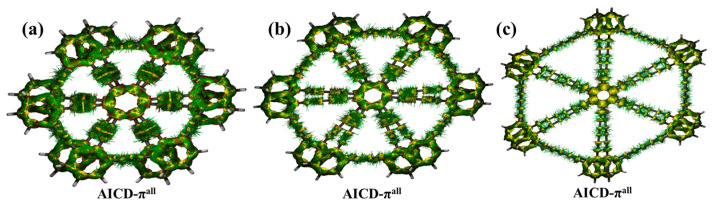
(**a**) AICD map of π electrons in bilayer graphyne; (**b**) AICD map of π electrons in bilayer graphdiyne; (**c**) AICD map of π electrons in bilayer graphtriyne (isovalue: 0.025 a.u).

The AICD function provides an effective tool for studying the anisotropy and aromaticity of molecules. It is applicable to planar molecular rings and can be extended to non-planar and non-closed systems. Current density maps, such as AICD plots, typically drawn on the molecular surface, are effective in distinguishing between different types of compounds, including aromatic and antiaromatic compounds [30]. Figure 6a shows that under a perpendicular magnetic field, the π-system of the benzene rings in bilayer graphyne generates a significant clockwise current, primarily distributed above and below the benzene rings, indicating aromaticity. The distribution of AICD-π^all^ further confirms this conclusion, consistent with the results of IRI and LOL analyses. Similarly, in Figure 6b, bilayer graphdiyne generates a comparable current under the same conditions, also demonstrating aromatic characteristics. As shown in Figure 6c, the AICD plot of bilayer graphtriyne exhibits a clockwise current within the benzene rings, though no global delocalized current is formed along the C–C bonds, still indicating aromaticity. By comparing the current density distributions in the AICD plots, it can be observed that as the number of acetylenic linkages increases, the current intensity decreases, and the overall aromaticity gradually diminishes.

### 2.7. Isoelectronic Shielding Surface (ICSS) Analysis

**Figure 7:**
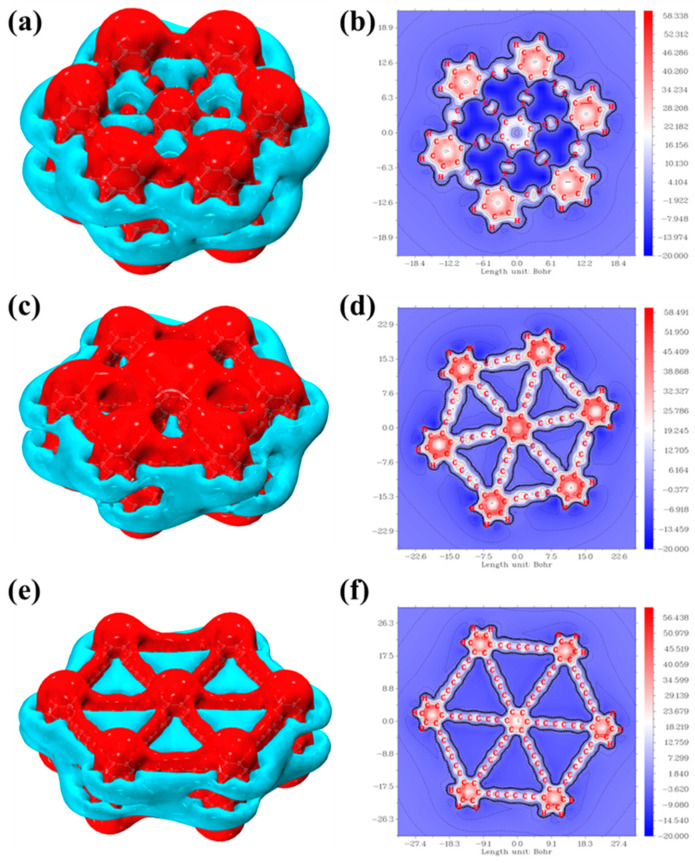
(**a**) ICSSZZ of bilayer graphyne (region color code for shielding zone: red, unshielded zone: blue) on an equivalent surface in a plane parallel to the ring (contour: 2.5); (**b**) color-filled map of ICSSZZ of bilayer graphyne (color bar scale in ppm); (**c**) ICSSZZ of bilayer graphdiyne; (**d**) color-filled map of ICSSZZ of bilayer graphdiyne; (**e**) ICSSZZ of bilayer graphtriyne; (**f**) color-filled map of ICSSZZ of bilayer graphtriyne. In the ICSSZZ color map, red represents the shielding zone, and blue represents the deshielding zone.

The isochemical shielding surface (ICSS) [31] is a real-space function closely related to the Nuclear Independent Chemical Shift (NICS) and can be used for the quantitative analysis of aromaticity in cyclic molecules. The ICSS calculates the magnetic shielding tensor at uniformly distributed grid points around the system and displays isosurface plots for specific components, clearly revealing the magnetic shielding or deshielding effects of delocalized electrons in different regions. The ICSSzz value represents the shielding effect of the system in the direction perpendicular to the molecular plane under an external magnetic field. When shielding or deshielding occurs at a specific point due to the external magnetic field, the local ICSSzz value at that point will show a positive or negative value. Figure 7a shows the ICSS isosurface of bilayer graphyne, where the red regions represent shielding effects and the blue regions represent deshielding effects. The shielding region in the vertical direction is significant, indicating that the benzene ring and C–C bond regions exhibit strong magnetic shielding effects, reflecting the pronounced aromaticity of the system. This shielding phenomenon arises because the magnetic induction currents generated by the π electrons do not fully cancel the external magnetic field within the benzene ring and C–C bond regions. In the C–H bond region, local induction currents generated by σ-electrons also shield the external magnetic field, resulting in the appearance of red isosurface contours around the C–H bond region. Figure 7b shows the color-filled ICSS map, highlighting high magnetic shielding values in the Z direction around the benzene ring in the molecular plane and demonstrating a smooth transition of magnetic shielding from the benzene ring to the C–C bonds, further revealing the aromaticity of bilayer graphyne. Figure 7c,d display the ICSS isosurface and color-filled map for bilayer graphdiyne. In the direction perpendicular to the molecular plane, the shielding regions remain significant, with strong magnetic shielding effects in the benzene ring and C–C bond regions, consistent with the aromaticity of this structure. Due to the magnetic induction currents generated by π electrons not fully canceling the external magnetic field in the benzene ring and C–C bond regions, the area around the C–H bonds is also locally shielded by these currents, resulting in surrounding red isosurfaces. The ICSS color-filled map confirms the aromaticity of bilayer graphdiyne. Figure 7e,f show the ICSS isosurface and color-filled map for bilayer graphtriyne. In the vertical direction, the shielding regions remain significant, with strong magnetic shielding effects observed in the benzene ring and C–C bond regions, indicating that this structure still possesses aromaticity. Although the magnetic induction currents of π electrons cannot fully cancel the external magnetic field in the benzene ring and C–C bond regions, the C–H bond regions are also locally shielded, further indicating the aromaticity of bilayer graphtriyne. By comparing the ICSSzz values and the distribution intensities in the color-filled maps, it can be observed that as the number of alkyne groups increases in bilayer graphyne, its aromaticity gradually weakens.

### 2.8. Optical Properties: UV–Visible Absorption Spectroscopy Analysis

**Figure 8:**
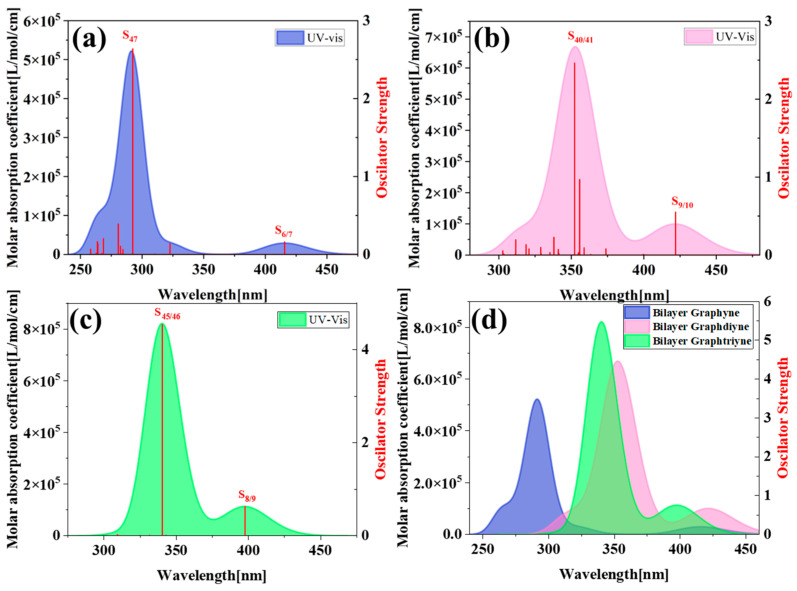
(**a**) UV-vis spectrum of bilayer graphyne; (**b**) UV-vis spectrum of bilayer graphdiyne; (**c**) UV-vis spectrum of bilayer graphtriyne. (**d**) Combined UV-vis spectra of bilayer graphyne, bilayer graphdiyne, and bilayer graphtriyne.

The ultraviolet–visible (UV-Vis) spectrum can reveal the transition energies and corresponding oscillator strengths of molecules from the ground state to various electronically excited states. In this study, the UV-Vis spectra of bilayer graphyne, bilayer graphdiyne, and bilayer graphtriyne were plotted, with each transition peak broadened into a Gaussian distribution, as shown in Figure 8. In the UV-Vis spectrum of bilayer graphyne, two significant absorption peaks appear in the wavelength range of 240–500 nm. The strongest absorption peak at 292.5 nm mainly originates from the contribution of the S_47_ excited state, while a weaker absorption peak at 415.7 nm is primarily contributed by the S_6_ excited state. For bilayer graphdiyne, the UV-Vis spectrum exhibits three absorption peaks within the range of 250–500 nm. The strongest peak at 352.3 nm is mainly attributed to the S_40_ excited state, whereas the weaker peak at 421.9 nm is predominantly contributed by the S_10_ excited state. In the UV-Vis spectrum of bilayer graphtriyne, two absorption peaks are observed in the wavelength range of 280–500 nm. The strongest peak at 340.3 nm is attributed to the S_45_ excited state, while the weaker peak at 397.6 nm primarily originates from the S_8_ excited state. These UV-Vis spectral features reveal the specific absorption behavior of different bilayer graphyne derivatives in the ultraviolet–visible region, reflecting the primary contributions of various excited states to the absorption peaks. The differences in the relative intensities and contributions of different excited states highlight the significant variations in electronic transition characteristics and light absorption properties among the derivatives.

### 2.9. Transition Density Matrix and Charge Difference Density Plot Analysis

**Figure 9:**
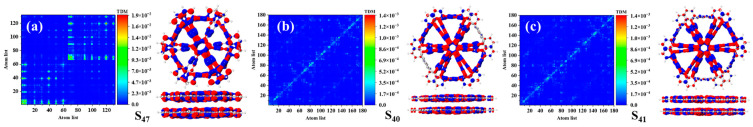
(**a**) The top view and front view of the TDM and CDD plots of bilayer graphyne in S_47_; (**b**) the top view and front view of the TDM and CDD plots of bilayer graphdiyne in S_40_; (**c**) the top view and front view of the TDM and CDD plots of bilayer graphtriyne in S_41_. In the CDD diagram, blue represents holes, while red represents electrons.

**Figure 10:**
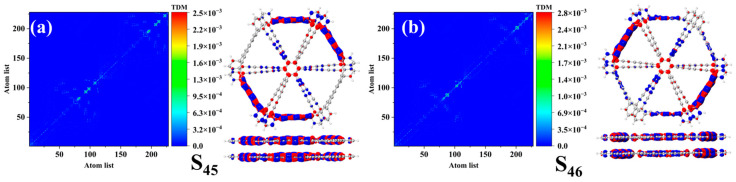
(**a**) The top view and front view of the TDM and CDD plots of bilayer graphtriyne in S_45_; (**b**) the top view and front view of the TDM and CDD plots of bilayer graphtriyne in S_46_. In the CDD diagram, blue represents holes, while red represents electrons.

The two-dimensional real-space analysis method, based on the representation of the transition density matrix in positional space, provides insights into electron transition behavior and electron–hole coherence in excited states. In the two-dimensional real-space coordinates, the charge transfer probability can be concretized through the horizontal and vertical coordinates of the data points. The charge difference density (CDD) plots reveal the locations of electrons and holes, corresponding to the regions of increased and decreased charge during electronic transitions. From the transition density matrix (TDM) of bilayer graphyne in the excited states (Figure 9 and Figure 10), it can be observed that the transition density of the S_47_ excited state is not only prominent along the diagonal but also exhibits significant electronic excitation features in the upper-left and lower-right regions. In the top view of the CDD plot for S_47_, the central carbon–carbon bonds are surrounded by blue isosurfaces, indicating that this region involves electronic excitation within the π orbitals. Similarly, the transition densities of the S_40_ and S_41_ excited states in bilayer graphdiyne are prominently distributed along the diagonal and exhibit electronic excitation features in the upper-left and lower-right regions. In contrast, the S_45_ and S_46_ excited states of bilayer graphtriyne display a transition density concentrated primarily along the diagonal. Overall, the transition density matrices for multiple excited states are surrounded by green isosurfaces in the diagonal regions, indicating minimal charge transfer and demonstrating weak local excitation characteristics. By analyzing the charge difference density (CDD) plots, it can be observed that the positions of electrons and holes alternate on the carbon rings under different excited states, further revealing the local excitation features of bilayer graphyne, bilayer graphdiyne, and bilayer graphtriyne.

### 2.10. Molecular Electrostatic Potential Analysis

**Figure 11:**
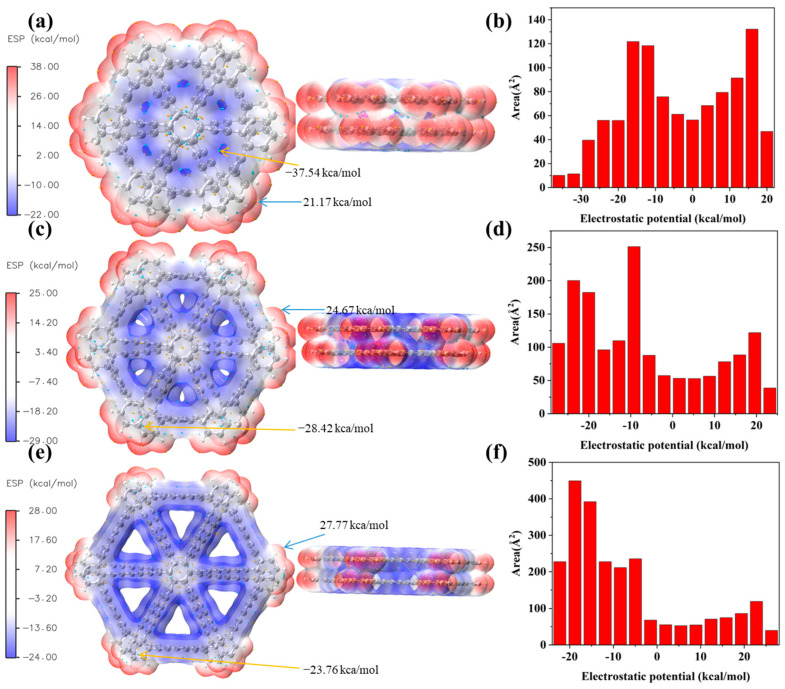
(**a**,**c**,**e**) Show the electrostatic potential (ESP) mapping on the van der Waals surface (i.e., the isosurface at ρ = 0.001 a.u.) for bilayer graphyne, bilayer graphdiyne, and bilayer graphtriyne, respectively. The redder the color, the more positive the ESP. The local minima and maxima on the ESP surface are represented by cyan and orange spheres, respectively. (**b**,**d**,**f**) Illustrate the area distribution across different ESP ranges for bilayer graphyne, bilayer graphdiyne, and bilayer graphtriyne, respectively.

The study of electrostatic potential (ESP) on the van der Waals (vdW) surface is often of interest, as it is closely related to intermolecular electrostatic interactions [32,33]. To predict the interactions between bilayer graphyne, bilayer graphdiyne, and bilayer graphtriyne with other chemical species due to electrostatic effects, we performed molecular surface analysis to identify the positions of the ESP extrema on their vdW surfaces, as shown in Figure 11a,c,e. Additionally, color maps illustrating the electrostatic potential distribution on their vdW surfaces were generated. Additionally, we calculated the area distribution of different ESP intervals on the vdW surfaces and presented them in Figure 11b,d,f. Two important conclusions can be drawn from these figures. Based on the values labeled in Figure 11a,c,e, the vdW surface ESP range of bilayer graphyne is −37.54 to 21.17 kcal/mol. In contrast, the vdW surface ESP range of bilayer graphdiyne is −28.42 to 24.67 kcal/mol, and that of bilayer graphtriyne is −23.76 to 27.77 kcal/mol. This indicates that as the number of acetylene groups increases, it becomes more challenging to form strong electrostatic interactions. Although the maximum ESP values on the surface are relatively high, the proportion of the surface area with positive ESP is smaller than that with negative ESP, as shown in Figure 11b,d,f. These visualizations provide insights into the shielding and deshielding effects across different regions of the bilayer structures.

## 3. Materials and Methods

In this study, the geometric structures of bilayer graphyne, bilayer graphdiyne, and bilayer graphtriyne were optimized and analyzed in the gas phase at the ωb97xd/6-311G(d,p) theoretical level, along with excited-state calculations [34]. The calculations using Density Functional Theory (DFT) and Time-Dependent Density Functional Theory (TD-DFT) were performed with the Gaussian 16 (A.03) program [35]. The wavefunctions obtained from theoretical calculations were analyzed using Multiwfn 3.8 (dev) [36], and the total density of states (TDOS) and projected density of states (PDOS) curves were plotted using Origin 2022 software. In addition, isosurface images of the interaction region indicator (IRI-π), localized orbital locator (LOL-π), and isoscalar chemical shielding surface (ICSSzz) were generated using VMD [37]. The AICD [38] code was used to analyze the anisotropy of the induced current density (AICD), and visualization images were generated using POV-Ray 3.7 [37]. The ultraviolet–visible absorption spectrum and transition density matrix (TDM) spectrum were plotted using Origin. In addition, VMD was used to generate charge density difference (CDD) images to analyze the optical properties. To ensure clear visualization, the molecules enclosed by all isosurfaces were placed in the xy plane.

## 4. Conclusions

In this study, the electronic structures and aromaticity characteristics of bilayer graphyne, bilayer graphdiyne, and bilayer graphtriyne were systematically investigated using various analytical methods, including molecular orbitals (MOs), the density of states (DOS), the interaction region indicator (IRI), the localized orbital locator (LOL), the anisotropy of the induced current density (AICD), and the isochemical shielding surface (ICSS). The results indicate that the number of acetylene chains significantly affects aromaticity; the greater the number of acetylene chains, the weaker the aromaticity. Specifically, bilayer graphyne demonstrates the least propensity for transitions, whereas bilayer graphdiyne exhibits the most facile transitions, suggesting changes in their electronic transition characteristics. Meanwhile, IRI and LOL analyses further confirm that the π electron delocalization in bilayer graphyne is significant; however, as the number of acetylene chains increases, electron delocalization gradually weakens, leading to reduced aromaticity. Additionally, AICD and ICSS analyses reveal the induced current and magnetic shielding properties of materials with different acetylene chains under an external magnetic field. Bilayer graphyne exhibits stronger aromatic characteristics, while bilayer graphtriyne displays weaker aromaticity. The UV-Vis spectra of bilayer graphyne, bilayer graphdiyne, and bilayer graphtriyne reveal significant differences in the contributions of various excited states to the absorption peaks. Bilayer graphyne exhibits notable absorption peaks at 292.5 nm and 415.7 nm, primarily contributed by the S_47_ and S_6_ excited states, respectively. Bilayer graphdiyne shows absorption peaks at 352.3 nm and 421.9 nm, which are mainly attributed to the S_40_ and S_10_ excited states. For bilayer graphtriyne, the absorption peaks at 340.3 nm and 397.6 nm are predominantly contributed by the S_45_ and S_8_ excited states. The transition density matrix indicates that these excited states exhibit density concentration in the diagonal regions, reflecting local excitation characteristics. The charge difference density (CDD) plots reveal an alternating distribution of electrons and holes on the carbon rings, further indicating the local excitation tendency of the bilayer structures. Meanwhile, as the number of acetylene groups increases, the van der Waals (vdW) surface electrostatic potential (ESP) range of bilayer graphyne, bilayer graphdiyne, and bilayer graphtriyne gradually narrows, suggesting a reduced ability to form strong electrostatic interactions. Additionally, the proportion of surface area with positive ESP is smaller than that with negative ESP.

## Data Availability

The original contributions presented in this study are included in the article/Supplementary Material. Further inquiries can be directed to the corresponding authors.

## Supplementary Materials

The following supporting information can be downloaded at: https://www.mdpi.com/article/10.3390/molecules30020365/s1, Figure S1: (a) The top view and front view of the TDM and CDD plots of bilayer graphyne in S6; (b) The top view and front view of the TDM and CDD plots of bilayer graphyne in S7; (c) The top view and front view of the TDM and CDD plots of bilayer graphyne in S47.; Figure S2: (a) The top view and front view of the TDM and CDD plots of bilayer graphdiyne in S9; (b) The top view and front view of the TDM and CDD plots of bilayer graphdiyne in S10; (c) The top view and front view of the TDM and CDD plots of bilayer graphdiyne in S40; (d) The top view and front view of the TDM and CDD plots of bilayer graphdiyne in S41.; Figure S3: (a) The top view and front view of the TDM and CDD plots of bilayer graphtriyne in S8; (b) The top view and front view of the TDM and CDD plots of bilayer graphtriyne in S9; (c) The top view and front view of the TDM and CDD plots of bilayer graphtriyne in S45; (d) The top view and front view of the TDM and CDD plots of bilayer graphtriyne in S46.
