# Supplementary material for: Determine the Relative Aromaticity of Bilayer Graphyne, Bilayer Graphdiyne, and Bilayer Graphtriyne
Source: Molecules. 2025 Jan 17;30(2):365. doi: 10.3390/molecules30020365 (PMC11767393; doi:10.3390/molecules30020365)
Supplement: Supplementary file 1 [file molecules-30-00365-s001.zip › molecules-3347373 - supplementary.pdf]

SUPPORTING INFORMATION

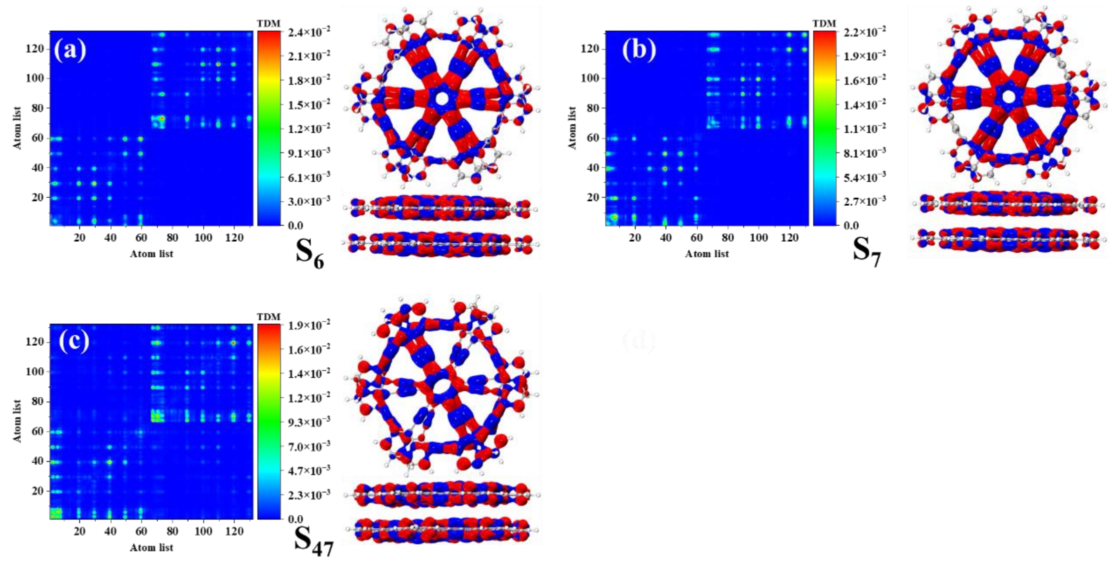

Figure S1. (a) The top view and front view of the TDM and CDD plots of bilayer graphyne in S_6;(b) The top view and front view of the TDM and CDD plots of bilayer graphyne in S_7;(c) The top view and front view of the TDM and CDD plots of bilayer graphyne in S_{47}.

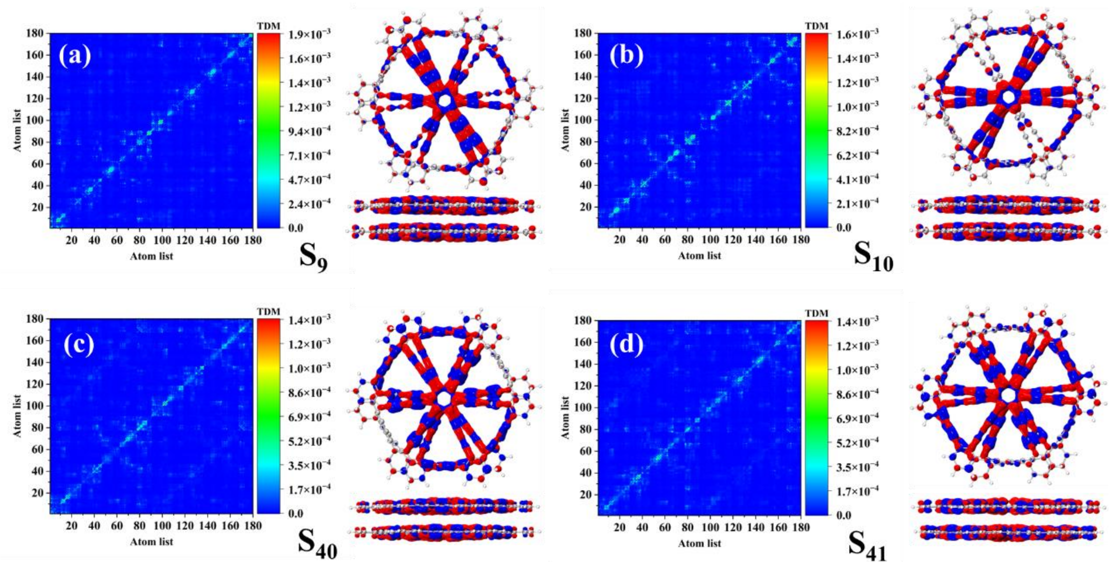

Figure S2. (a) The top view and front view of the TDM and CDD plots of bilayer graphdiyne in S_9;(b) The top view and front view of the TDM and CDD plots of bilayer graphdiyne in S_{10};(c) The top view and front view of the TDM and CDD plots of bilayer graphdiyne in S_{40}; (d) The top view and front view of the TDM and CDD plots of bilayer graphdiyne in S_{41}.

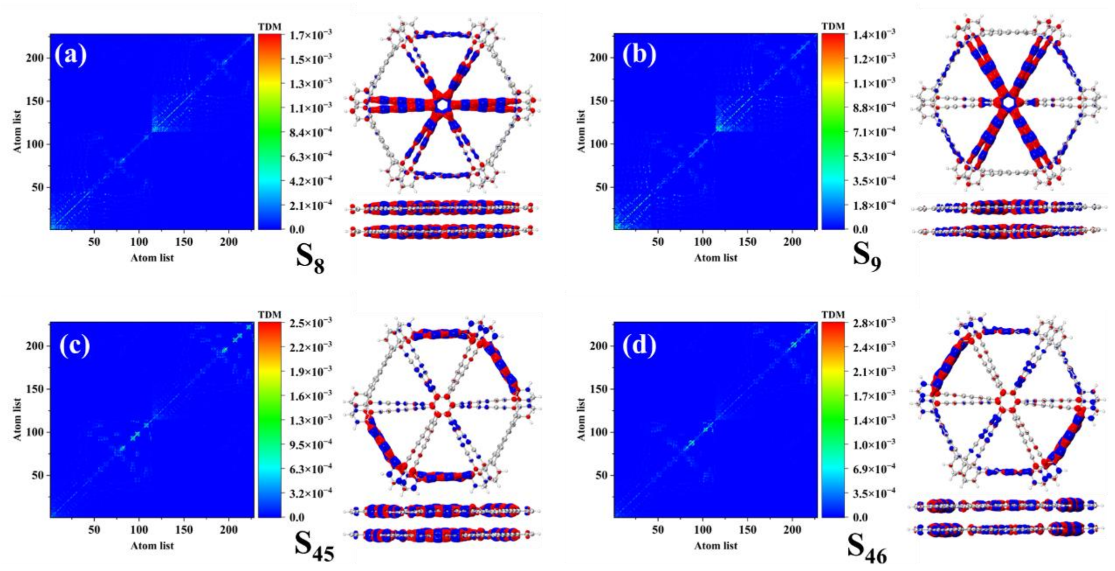

Figure S3. (a) The top view and front view of the TDM and CDD plots of bilayer graphtriyne in S_8;(b) The top view and front view of the TDM and CDD plots of bilayer graphtriyne in S_9;(c) The top view and front view of the TDM and CDD plots of bilayer graphtriyne in S_{45}; (d) The top view and front view of the TDM and CDD plots of bilayer graphtriyne in S_{46}.
